# A biomechanical analysis of triangular medial knee reconstruction

**DOI:** 10.1186/s12891-018-2039-1

**Published:** 2018-04-20

**Authors:** Xiaomeng Wang, Huixin Liu, Guman Duan, Yingzhen Niu, Chang Liu, Fei Wang

**Affiliations:** Department of Orthopaedic Surgery, Third Hospital of Hebei Medical University, No.139 Ziqiang Road, Shijiazhuang, 050051 Hebei China

**Keywords:** Superficial medial collateral ligament, Posterior oblique ligament, Triangular reconstruction, Anatomical reconstruction, Stability

## Abstract

**Background:**

The purpose of this study was to evaluate and compare knee kinematics and stability following either triangular or anatomical reconstruction of the superficial medial collateral ligament (sMCL) and posterior oblique ligament (POL).

**Methods:**

In a cadaveric model (12 knees), the stability and kinematics following two experimental sMCL and POL reconstructions were compared in sMCL- and POL-deficient knees versus normal knees. The first reconstruction was a triangular reconstruction of the sMCL and POL, while the second involved an anatomical reconstruction of the sMCL and POL. All knees were tested through four different states. The changes in valgus angles, external rotation, and internal rotation were measured in the normal and sMCL- and POL-deficient knees, as well as in the knees that had undergone the two different forms (triangular and anatomical) of reconstruction.

**Results:**

After initial sectioning of the sMCL and POL, we observed significantly increased valgus rotation, external rotation, and internal rotation at all knee flexion angles (0°, 20°, 30°, 60°, 90°). Additionally, passive stability testing demonstrated a significant increase in tibial internal rotation following triangular reconstruction compared with anatomical reconstruction at knee flexion angles of 20° and 30°. A significant increase in internal rotation was present following triangular reconstruction compared with anatomical reconstruction at 20° (mean difference = 2.77) (*P* = 0.008) and 30° (mean difference = 0.99) (*P* < 0.001) of knee flexion.

**Conclusion:**

This study suggests that anatomical sMCL and POL reconstruction produces slightly better biomechanical stability than triangular reconstruction. However, triangular reconstruction may restore a near-normal knee joint is both less invasive and more practical.

## Background

Isolated medial collateral ligament (MCL) injury often does not require surgical treatment [1–3]. However, severe MCL injuries are often accompanied by injury to the posteromedial structures of the knee [4]. Halinen [5] and Indelicato [3] suggested that injuries to the posteromedial structures can heal with conservative treatment, but they may result in residual joint laxity and increased rotation, which can lead to osteoarthritis and eversion in the long term [6]. Thus, early surgery should be considered in patients with severe posteromedial structure injuries [7].

The posteromedial structures are extremely important to valgus and rotational stability of the knee. The superficial MCL (sMCL) plays a major role in valgus stability of the knee joint, and a secondary role in internal and external rotation stability [8, 9]. The posterior oblique ligament (POL) has an important function in rotational stability, and a secondary function in valgus and external rotation stability [10–12]. From a biomechanical point of view, the sMCL contributes 78% of the stability in valgus and external rotation at 25° of knee flexion, and the POL plays a primary role in internal rotation and preventing valgus when knee flexion is between 0°~ 30° [8, 13]. Thus, it is clear that repairing the sMCL and POL efficiently simultaneously plays a dominant role in the stability and biomechanics of the knee joint [6].

Slocum [14], Hughston [15], and Fanelli [16] highlighted the importance of tight suturing of the posteromedial capsule and the POL when reconstructing the sMCL. Lind [17], Coobs [18], and Azar [19] both described operative methods for double-bundle reconstruction of the sMCL and POL in treating chronic damage to the posteromedial structures of the knee. Coobs [18] confirmed in an in vitro biomechanical study that normal medial knee stability can be restored by reconstructing the sMCL and POL at their anatomical attachment points. Feeley confirmed that reconstructing the sMCL based on its anatomical footprint led to the smallest biomechanical variation [20]. Thus, the consensus has been that anatomical reconstruction of the sMCL is the optimal approach. The POL is an important ligament as well, but the effects of changing its attachment point are not well understood.

With anatomical reconstruction of the sMCL and POL, four tunnels, four interface screws, and two ligaments are used [18]. The triangular reconstruction method was created based on traditional clinical operative methods. Two femoral bone tunnels and two tibial bone tunnels are each substituted with one tunnel. The outcomes following use of this method have not been well demonstrated in experimental or clinical studies.

The purpose of our study was to compare the stability and kinematics following either triangular or anatomical reconstruction of the sMCL and POL in a cadaveric model. We hypothesized that the triangular technique would yield similar efficacy compared with anatomical reconstruction of the sMCL and POL.

## Methods

### Specimen materials

Twelve fresh-frozen cadaveric knees were used in the current study. Specimens with ligamentous laxity, severe arthritis, or previous surgery were excluded. The cadaveric knees averaged 51.3 years of age, and included eight males and four females, three left knees, five right knees. All of the specimens were provided by the third Affiliated Hospital of Hebei Medical University. Informed consent to participate in the study was obtained from the patients, and written consent was obtained from the patients before they died to use their knees after death. All knee joints were thawed 24 h before being processed. The femur and tibia were removed 25 cm above and below the joint line, and then were fixed together with a steel wire.

### Testing methods

**Fig. 1:**
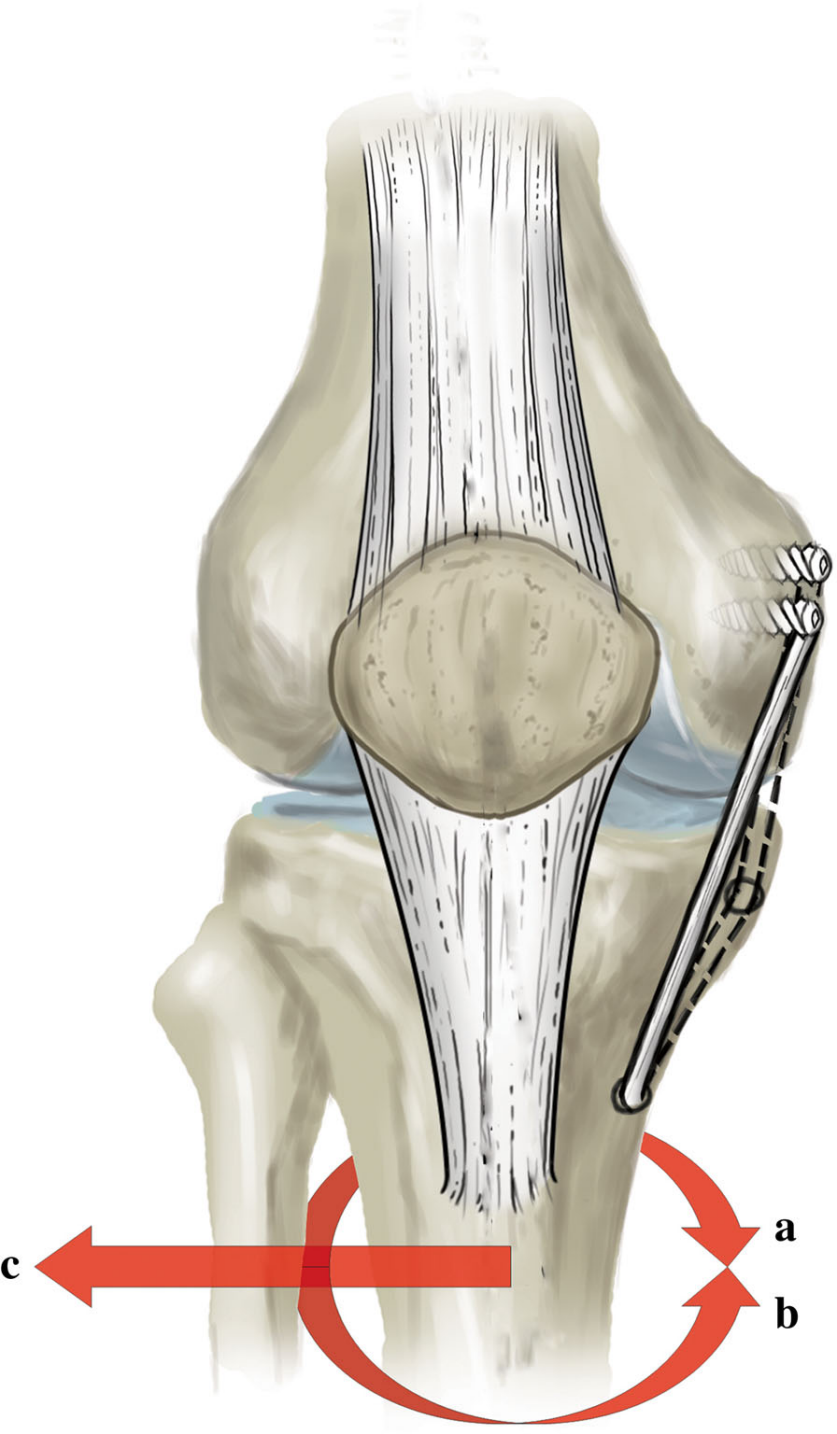
The schematic diagram of motion process. **a** external rotation **b** internal rotation **c** valgus movement

All knees were tested in four different states. The changes in valgus, external rotation, and internal rotation were measured in normal and sMCL- and POL-deficient knees, as well as in those that had undergone triangular and anatomical reconstructions (valgus moment of 10 N.m, internal rotation of 5 N.m, and external rotation moments were applied to the knee) [18] [Fig. 1] at 0°, 20°, 30°, 60°, and 90° of knee flexion using a BOSE biomechanical instrument (3520-AT, System 100306, Eden Prairie, MN, USA). Four different types of knees (intact, deficient sMCL and POL, triangular reconstruction, and anatomical reconstruction) were compared in this study. The knees were first tested in the intact state, then tested in the deficient state, then followed by the triangular reconstruction and anatomical reconstruction states. Biomechanical testing was repeated to measure the changes in angles in the four different states.

### Allogeneic tendon preparation

One allogeneic tendon was thawed in physiological saline for 20 min, and its length and diameter were measured. (Length ≥ 20 cm, diameter 4–5 mm). A 2.5-cm lock-stitch suture was made at the thicker end of allogeneic tendon with No. 1 suture (Ethibond Excel 4.0 metric). 2-0 Ethicon Vicryl was prepared for use as a pull wire after measuring the diameter of the sutured tendon. A 2.5-cm lock-stitch suture was made at each portion of allogeneic tendon with No. 1 suture (Ethibond Excel 4.0 metric) [18].

### Triangular reconstruction technique

**Fig. 2:**
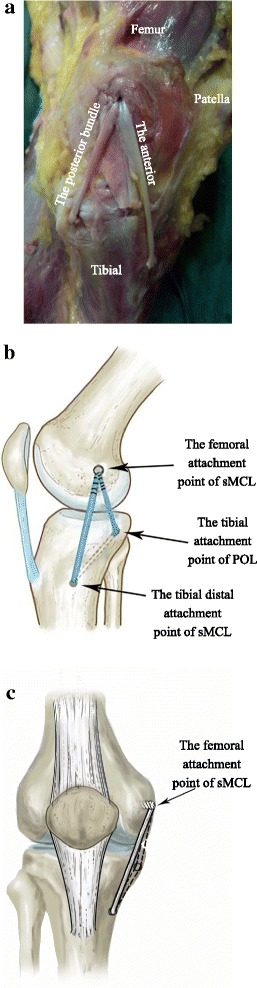
Triangular reconstruction of the sMCL and POL. The femoral attachment site of the reconstructed sMCL is the sMCL anatomical attachment point. The femoral attachment site of the reconstructed POL is the sMCL anatomical attachment point. The tibial fixation points are the distal sMCL and POL anatomical attachment points. The reconstructed ligament forms a triangle. **a** The medial view of the experimental image. **b** Schematic diagram viewed from the side. **c** Schematic diagram viewed from the front

A medial incision was made from the medial femoral epicondyle to the medial proximal tibia to expose the entire MCL and POL. The femoral and tibial anatomical attachment points of the sMCL and POL were exposed by blunt dissection. The sMCL has two attachment points on the tibial side, one located about 1 cm away from the joint line, and another located about 6 cm away from the joint [21]. The anterior bundle was the distal tibial attachment of the sMCL; the posterior bundle was the tibial attachment of the POL [21]. We flexed the knee 30° and used an anterior cruciate ligament reconstruction tibial locator to locate the tibia accurately. We then threaded an eyelet pin as a hook pin from the center of the sMCL tibial insertion to the POL tibial insertion, and used a reamer (4.5 mm) matched in diameter with the allograft tendon to drill the tibial tunnel along the eyelet pin. After that, we pulled the tendon through the tibial tunnel using a traction wire and threaded an eyelet pin from inside to outside at the sMCL femoral attachment point of the medial femoral condyle. We used the eyelet pin location to determine the length of tendon and wipe off redundant tendon, and then weaved the other side of the tendon 2.5 cm and continuously sutured the traction wire. We measured the diameter of both distal tendons and selected a reamer (7 mm) to match with it to drill the femoral tunnel to a depth of 30 mm. We threaded both ends of the tendon under the sartorius tendon and pulled them into the femoral tunnel of the sMCL, and then set the position of the tendon and confirmed that the two beams of the tendon had enough tension while pulling traction on both ends. We flexed the knee joint to 30° [20], and inserted a bioabsorbable screw tap into the femoral tunnel that matched the diameter of the tunnel (Fig. 2).

### Anatomical reconstruction technique

**Fig. 3:**
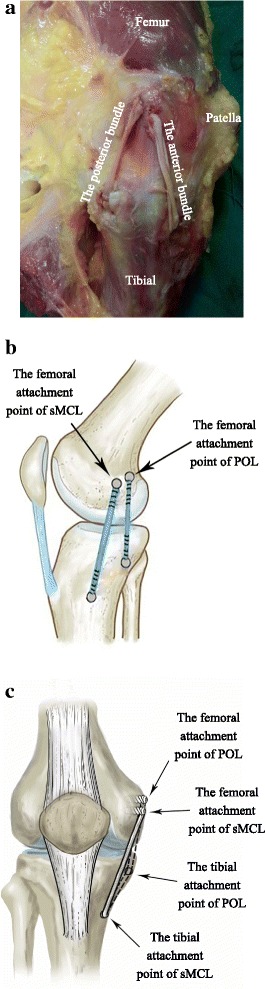
Anatomical reconstruction sMCL and POL. The femoral attachment sites of the anatomical reconstruction are the sMCL and POL anatomical attachment points. The tibial fixation points are the distal sMCL and POL anatomical attachment points. All reconstructed sites are anatomical sites. **a** The medial view of the experimental image. **b** Schematic diagram viewed from the side. **c** Schematic diagram viewed from the front

The anatomical reconstruction technique of the sMCL and POL has previously been described in an article by LaPrade and colleagues [21]. The sMCL and POL were reconstructed with four reconstruction tunnels and four interference screws [18]. In our experiment, three tunnels and two interface screws were used to anatomically reconstruct the sMCL and POL (Fig. 3).

### Statistical analysis

Data were collected using a biomechanical test instrument for measuring the angles of knee extroversion and tibial rotation at different angles of flexion. The results are presented as mean ± standard deviation. Data processing was performed using SPSS 21.0 (SPSS Inc., Chicago, USA), and the analyses were performed using two-factor analysis of variance. Tests were performed at each knee state (0°, 20°, 30°, 60°, and 90° of flexion) and measurement index. Factor one was the specimen (nine levels) and factor two was the treatment (four levels). The level of significance was defined as *P* < 0.05.

## Results

The biomechanical results of this experiment are listed in Tables 1, 2 and 3.

**Table 1:** Valgus angulation with the application of an externally applied load for each testing state

Flexion angles	0°	20°	30°	60°	90°
Intact	3.11 ± 0.49	5.58 ± 1.49	7.53 ± 1.40	9.47 ± 1.26	10.37 ± 1.12
Deficient	8.89 ± 1.22*	15.83 ± 2.20*	16.61 ± 1.68*	17.87 ± 1.14*	17.50 ± 1.18*
Triangular	5.87 ± 0.35*	6.50 ± 2.14	7.76 ± 1.02	9.81 ± 0.80	10.29 ± 1.24
Anatomical	5.90 ± 0.33*	6.19 ± 1.86	7.43 ± 1.09	9.18 ± 0.80	9.78 ± 0.79

Values are presented as mean ± standard error of the mean

*Significant difference compared with the Intact group (*p* < 0.05)

**Table 2:** External rotation with the application of an externally applied load for each testing state

Flexion angles	0°	20°	30°	60°	90°
Intact	9.64 ± 0.88	14.36 ± 1.91	14.74 ± 0.88	16.00 ± 0.72	18.34 ± 1.41
Deficient	13.18 ± 0.99*	20.30 ± 2.01*	20.95 ± 0.58*	25.91 ± 0.76*	29.03 ± 0.48*
Triangular	9.97 ± 1.00	15.32 ± 2.06	15.34 ± 1.06	16.55 ± 0.48	18.18 ± 1.07
Anatomical	10.13 ± 0.51	16.28 ± 3.32	14.70 ± 1.08	16.61 ± 1.00	17.93 ± 1.06

Values are presented as mean ± standard error of the mean

*Significant difference compared with the Intact group (*p* < 0.05)

**Table 3:** Internal rotation with the application of an externally applied load for each testing state

Flexion angles	0°	20°	30°	60°	90°
Intact	9.74 ± 0.31	16.46 ± 1.94	16.05 ± 0.35	7.53 ± 1.40	12.14 ± 0.47
Deficient	15.87 ± 0.46*	24.25 ± 2.19*	17.94 ± 0.44*	16.61 ± 1.68*	13.78 ± 0.74*
Triangular	10.13 ± 0.74	19.23 ± 2.81*	15.95 ± 0.58*^§^	7.76 ± 1.02	12.46 ± 0.55
Anatomical	9.83 ± 0.29	17.27 ± 2.76	16.05 ± 0.45	7.43 ± 1.09	12.61 ± 0.87

Values are presented as mean ± standard error of the mean

*Significant difference compared with the Intact group (*p* < 0.05)

^§^Significant difference compared triangular groups with the anatomical group (*p* < 0.05)

### Valgus rotation

**Fig. 4:**
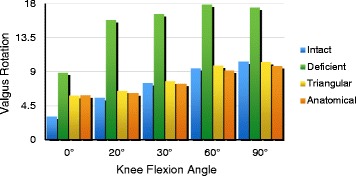
The changes in the knee joint of each group were observed under a force of 10 N.m. After initial sectioning of the sMCL and POL, we observed significantly increased valgus rotation at all knee flexion angles. In addition, a significant decrease was observed after sMCL and POL reconstruction compared with the deficient sMCL and POL states at all knee flexion angles. A significant increase was observed in valgus angulation of the anatomically reconstructed knee compared with the intact reconstruction at 0° of knee flexion. The mean valgus angulation with the triangular reconstruction was significantly different from the normal knee at 0° of knee flexion

The changes in the knee joint of each group were observed under a force of 10 N.m. After initial sectioning of the sMCL and POL, we observed significantly increased valgus rotation at all knee flexion angles (0°, 20°, 30°, 60°, 90°). In addition, a significant decrease was observed after sMCL and POL reconstruction compared with the deficient sMCL and POL states at all angles of knee flexion. The mean values of the reconstructed knees were not significantly changed compared with the normal knee at 20°, 30°, 60°, 90° of knee flexion (*P* < 0.05). There were no significant differences when comparing the intact with the triangular or anatomical reconstructions. A significant increase was observed in valgus angulation of the anatomically reconstructed knees compared with the intact reconstructed knees at 0° of flexion (mean difference = 2.79°) (*P*<0.001). The mean value (2.66°) of valgus angulation of the triangular reconstruction was significantly different than in the normal knee at 0° of knee flexion (*P* < 0.001). The mean values of valgus angulation of the triangular reconstruction were not significantly different from the anatomical reconstruction at 0°, 20°, 30°, 60°, and 90° of knee flexion. No significant differences were observed in tibial valgus rotation of the triangular reconstruction compared with the anatomical reconstruction at 0° (*P* = 0.908), 20° (*P* = 0.699), 30° (*P* = 0.540), 60° (*P* = 0.131), 90° (*P* = 0.255) (Fig. 4) (Table 1).

### External rotation

**Fig. 5:**
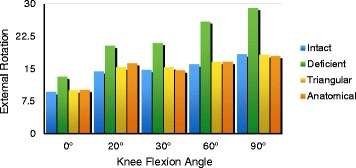
The changes in the knee joint of each group were observed under a force of 5 N.m. We found a significant increase in external rotation after sectioning the sMCL and POL at all tested knee flexion angles. In addition, a significant decrease was observed in knees with the sMCL and POL reconstruction compared with the sMCL- and POL-deficient knees at all angles of knee flexion

The changes in the knee joint of each group were observed under a force of 5 N.m. We found a significant increase in external rotation after sectioning the sMCL and POL at all tested knee flexion angles (0°, 20°, 30°, 60°, 90°). In addition, a significant decrease was observed in knees with sMCL and POL reconstruction compared with SMCL- and POL-deficient knees at all angles of knee flexion. The mean external rotation in the triangular or anatomical reconstruction knees were not significantly different than in the intact knee at 0°, 20°, 30°, 60°, and 90° of knee flexion (*P* < 0.05) (Fig. 5) (Table 2).

### Internal rotation

**Fig. 6:**
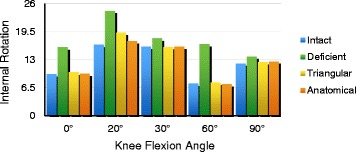
The changes in the knee joint of each group were observed under a force of 5 N.m. After initial sectioning of the sMCL and POL, we observed significantly increased internal rotation at all knee flexion angles (0°, 20°, 30°, 60°, 90°). A significant increase was observed in internal rotation of the triangular reconstruction compared with the intact reconstruction at knee flexion angles of 20° and 30°. The mean value of internal rotation with the anatomical reconstruction changed significantly compared with the normal knee at 30° of knee flexion

The changes in the knee joint of each group were observed under a force of 5 N.m. After initial sectioning of the sMCL and POL, we observed significantly increased internal rotation at all knee flexion angles (0°, 20°, 30°, 60°, 90°). Nevertheless, the mean values of internal rotation following triangular reconstruction were not significantly different compared with normal knees at 0°, 60°, and 90° of knee flexion (*P* < 0.05). A significant increase was observed in internal rotation of the triangular reconstruction knees compared with the intact reconstruction knees at 20° (mean difference = 2.77) (*P* = 0.008) and 30° (mean difference = 0.99) (*P* < 0.001) of knee flexion. The mean values of internal rotation following anatomical reconstruction were not significantly different than in the normal knees at 0°, 60°, and 90° of knee flexion (*P* < 0.05). The mean internal rotation (0.93°) with anatomical reconstruction was significantly different from the normal knee at 30° of knee flexion (*P* < 0.001) (Fig. 6) (Table 3).

## Discussion

The goal of the study was to perform a cadaveric experiment in order to evaluate the kinematics of patients undergoing either anatomical reconstruction or triangular reconstruction of the sMCL and POL. The most significant finding was that anatomical reconstruction restored the biomechanical stability of the sMCL- and POL-deficient knee. Compared with anatomical reconstruction, triangular reconstruction can largely restore the stability of the knee joint. There were small differences between anatomical reconstruction and triangular reconstruction, which can likely be ignored.

MCL injury is the most common ligament injury of the medial knee joint. Isolated sMCL injuries often heal with appropriate conservative treatment [3, 22]. However, they are frequently found to be accompanied by POL injuries [4, 15], and conservative management of these combined injuries may lead to functional limitations and osteoarthritis [6]. Therefore, simultaneous operative treatment of the sMCL and POL is necessary in some circumstances.

The MCL plays an important role in the movement of the knee joint (valgus, external rotation, and internal rotation), especially in valgus [12]. The POL is complementary to the MCL, and also plays a role in valgus [12, 23–25]. The valgus angles of the tested knees increased significantly after cutting off the MCL and POL. This experiment confirmed the important role of the sMCL and the POL in the valgus stability of the knee joint, as observed in previous studies [12, 17]. Studies have demonstrated that the sMCL provides 78% of the restraining force against valgus power at 25° of knee flexion [4, 8, 14]. Simultaneously, an obvious increase (10.25°) in valgus was observed at 20° of knee flexion with sectioning of the medial knee structures. The sMCL and POL also have important roles in rotational stability [12]. Sectioning of the sMCL and POL also resulted in a significant increase in external rotation and internal rotation of the tibia at several different knee flexion angles (0°, 20°, 30°, 60°, and 90°).

The recovery of anatomical structures and biomechanical function of the knee must be considered when creating a therapy program. An anatomically reconstructed knee has been shown to lead to biomechanics similar to that of a normal knee joint [18, 20, 26]. Our experimental model confirmed that anatomical reconstruction can not only effectively restore anatomical structures but can also effectively restore the biomechanics of the knee joint. There were no obvious differences between the intact and the anatomically reconstructed knees at knee flexion angles of 20°, 30°, 60°, and 90°. The results of this study are similar to those reported by Coobs [18]. Only partial valgus relaxation remained at 0° of knee flexion. The triangular reconstruction technique was performed due to the femoral attachments of the sMCL and the POL being in close proximity [21]. The femoral attachments of the sMCL and the POL were merged as a single reconstruction point (sMCL), and the tibial attachments of the sMCL and POL remained at their anatomical attachment points in this experimental model. In order to evaluate the effectiveness of triangular reconstruction of the sMCL and POL, this study tested static knee stability before and after ligament reconstruction, and compared the stability following the triangular and anatomical reconstructions compared with the normal knee. The triangular medial knee reconstruction technique provided a full recovery to native stability at 0°, 60°, and 90°of knee flexion. A small but significant increase was found in tibial internal rotation of the triangular reconstruction knee as compared with the intact knee (2.77° and 0.99° at 20° and 30° of knee flexion, respectively). Although we did find small differences in internal rotation at 20° and 30° of knee flexion, we believe these differences were not clinically significant. Compared with the anatomical reconstruction, a small difference (0.93°) in internal rotation was found at 30° of knee flexion. This was due to the significant role of the POL in internal rotation stability of the knee joint [10]. The majority of rotational stability was restored after triangular reconstruction. Furthermore, it also reduced the amount of hardware used in the internal fixation.

This study had inherent limitations. Our sample size was small, although it was powered to show statistical differences between groups. This experiment did not simulate muscle tension and only involved static biomechanical tests. Another limitation was that we did not verify the clinical effectiveness of this method.

## Conclusion

This study suggests that anatomical sMCL and POL reconstruction may produce slightly better biomechanical stability than the triangular reconstruction, but the triangular reconstruction may restore a near-normal knee joint, and is both less invasive and more practical.

## Abbreviations

MCL: Medial collateral ligament
POL: Posterior oblique ligament
sMCL: Superficial medial collateral ligament
